# Application of Failure Mode and Effect Analysis in Laparoscopic Colon Surgery Training

**DOI:** 10.1007/s00268-014-2827-1

**Published:** 2014-10-18

**Authors:** Francisco Alba Mesa, Miguel Angel Sanchez Hurtado, Francisco Miguel Sanchez Margallo, Virginia Gomez Cabeza de Vaca, Andrzej L. Komorowski

**Affiliations:** 1 Consorcio Sanitario Publico del Aljarafe, Hospital San Juan de Dios, Bormujos, Sevilla Spain; 2 Department of Laparoscopic Surgery, Minimally Invasive Surgery Centre Jesús Usón, Cáceres, Cáceres Spain; 3 Department of Surgical Oncology, Maria Skłodowska-Curie Memorial Cancer Centre and Institute of Oncology, ul.Garncarska 11, 31-115 Kraków, Poland

## Abstract

**Aim:**

To evaluate if application of failure mode and effect analysis (FMEA) to laparoscopy training can help surgeons acquire laparoscopy skills.

**Methods:**

After preparing a FMEA matrix of laparoscopic sigmoidectomy, we have introduced it during three laparoscopy courses. Forty-eight surgeons, divided into 24 teams of two surgeons, have participated in three courses. During each course, every team has performed three laparoscopic sigmoidectomies in three experimental animals (1 OR session every day). Risk priority number (RPN) has been calculated for every surgery, and the results have been discussed at the end of each training day with all participants.

**Results:**

We have observed a decline in the median RPN from 1339 during the first OR session through 62 during second OR session to reach 0 in the third OR session. Only two teams out of 24 were not able to reach a RPN of less than 300 during third OR session. When the type of failures were analysed, we have observed a shift from procedure-type failures to technical failures that depended on each participant technical abilities.

**Conclusion:**

Application of FMEA principles to laparoscopy training can help acquire non-technical skills necessary for safe laparoscopic surgery.

## Introduction

Advanced laparoscopy training is a process that includes theoretical basis and practical skills teaching [1]. Several studies have confirmed that simulation training in laparoscopy yields better results than non-simulation training [2]. It seems that abilities learned during simulation training are transferable to the operative room [3]. However, it is still the matter of debate what kind of simulation training yields better results [4]. Simulators may be the best way to start for a beginner laparoscopist, while for advanced students the use of animal models should probably be the last step before performing laparoscopy procedures on human patients. Up to 86 % of surgical residents see animal model as the best way to acquire laparoscopy skills [5].

Animal model is widely used as a platform for technical skills perfectioning. However, it can also be used for the evaluation of the ability of students to perform a safe operation. Among many tools used for the assessment of complex procedures, the failure mode and effect analysis (FMEA) is one of the most commonly used in the industry outside healthcare. FMEA is a method used already from the 60ties in the aerospace industry and later introduced in automobile industry. It uses a standardized approach to assess a complex process in order to identify the elements that carry a highest risk of harm and thus prioritise the measures used to protect from these risks [6].

The originality of the FMEA consists on prospective approach, i.e., it identifies possible errors before they occur. This approach enables each of the elements of a process to be attributed a cumulative numerical value (risk priority number—RPN) which is then used to prioritise the actions taken against this particular element. The numerical value rates the severity, probability and detectability of each failure mode. The potential of risk of each element of the process is calculated with the interpretation of three indexes that consider the severity of the event (SE: assesses the implications of the failure), probability of occurrence (PO: assesses the probability of the failure to appear in each step) and probability of detection (PD: assesses in each step the probability of detecting a failure). These indexes give rise to the RPN. This number is a value between 1 and 1000, with 1 being the minimum impact on the process and 1000 the maximum negative impact. It is calculated using the following formula: RPN = SE * PO * PD [7].

Traditional tools used for the evaluation of technical errors and events during laparoscopy training such as the objective structured assessment of technical skill (OSATS) are aimed at quantitative and qualitative evaluation of several tasks [8]. These methods try to measure objective surgical skills in the moment of action. FMEA on the other hand establishes an interpretation of potential failures and their future impact on the patient. Because of that, those two groups of methods (FMEA versus quantitative and qualitative methods such as OSATS) may not be comparable due to their metrics and aims. Although both focus on the improvement of surgical training, they should not be included in the same surgical skills assessment tools group because the former aims at non-technical evaluation and the latter aims at technical aspects of a process.

In particular, the FMEA as the evaluation tool has a prospective character and consists of the following:

- identification and evaluation of failure modes of a product or failure mode of a process and its consequences
- identification of actions that can eliminate the possibility of occurence of previously identified failure modes.
- documentation of the results

Although the FMEA is applied mainly in the phase of development of a process or a product, it is also valid to any complex situation. In this study, we have tried to apply the FMEA philosophy to laparoscopy training course using pigs as a model for laparoscopic sigmoidectomy.

## Methods

### FMEA matrix for the laparoscopic sigmoidectomy

We have applied FMEA methodology to laparoscopic sigmoidectomy training using pigs as an experimental model. Before starting the laparoscopic courses, we have designed a matrix for the process (Table 1) in which all phases of the process entitled “laparoscopic sigmoidectomy” have been defined. For each phase of the process, we have analysed the possibility of a preventable failure. For every failure, we have analysed possible consequences. The elements considered in the FMEA matrix as potential causes of failure were established in advance by an interdisciplinary working group participating in each step of the operation (surgeons, anaesthetist and nursing staff). Each element of the process (each surgical step) has been assigned a numerical value reflecting its severity (SE), probability (PO) and detectability (PD). The numerical value assigned to a particular element of the process was a result of the discussion between the members of the team. The whole team involved in creating the matrix for the process has been previously trained and educated in the FMEA method.

**Table 1:** Summarized matrix of the most representative phases for the process “laparoscopic sigmoidectomy” for each of which a RPN has been calculated as follows: RPN = SE * PO * PD

Phase	Possible failure	Reason of failure	Direct effect of failure	Indirect effect of failure	SE	PO	PD	RPN
1. Fixation of patient to the operation table	Failure to fix patient correctly	Ignoring the preparation step	Fall of patient from the operation table	Lesions caused by fall	7	5	9	315
			Problems in obtaining surgical field	Conversion to open surgery	8	5	1	40
			Peripheral nerve entrapment syndromes	Functional impairment	7	5	9	315
2. Trocars placement	Bleeding from the trocar site	Technical	Hemodynamic impairment	Transfusion	4	8	2	64
			Longer time to start surgery	Longer anaesthesia time	4	8	2	64
			Postoperative bleeding	Reoperation	8	8	6	384
	Wrong trocar placement	Technical	Longer surgery time	Longer anaesthesia time	4	8	2	64
		Erroneous tumour localization	Longer anaesthesia time	Longer anaesthesia time	4	8	2	64
			Conversion to open surgery	Longer anaesthesia time	7	8	1	56
				Rise in SSI	8	8	1	64
	Trocar site non-suitable for ileostomy	Technical	Infection of trocar site for ileostomy	Rise in SSI	8	8	1	64
3. Localisation and division of the arterial pedicle	Separate division of haemorrhoidal and sigmoidal arteries	Technical	Low number of lymph nodes	Oncological failure	8	8	9	576
	Bleeding	Technical	Conversion to open surgery	Rise in SSI	7	8	1	56
	Confounding haemorrhoidal artery with ureter	Technical	Longer surgery time	Longer anaesthesia time	7	8	1	56
			Ureter lesion	Functional impairment	8	8	6	384
4. Localization and dissection of the ureter	Inadvertent division of the ureter	Technical	Tutorization of the ureter	Longer anaesthesia time	7	8	1	56
		Confounding haemorrhoidal artery with ureter	Urethral stenosis	Functional impairment	8	8	6	384
		Confounding with gonadal vessels	Conversion to open surgery	Rise in SSI	7	8	1	56
5. Division of the surgical specimen	Intestinal wall perforation	Technical	Conversion to open surgery	Oncological failure	8	7	1	56
	Various sections of the specimen (many stapler loads)	Wrong trocar placement	Anastomotic dehiscence	Functional impairment	8	7	7	392
		Narrow pelvis	Anastomotic dehiscence	Functional impairment	8	7	7	392
		Small cutting linear stapler load	Anastomotic dehiscence	Functional impairment	8	7	7	392
6. Mechanical anastomosis	Twist of the proximal end	Technical	Anastomotic dehiscence	Functional impairment	8	3	8	192
	Impossibility to introduce circular stapler	Narrow pelvis	Longer surgery time	Longer anaesthesia time	7	8	1	56

RPN values range = 1–1000

*SE* severity of event, *PO* probability of occurence, *PD* probability of detection, *RPN* risk priority number, *SSI* surgical site infections

A RPN was calculated for each failure based on severity of event (SE), probability of occurence (PO) and probability of detection (PD) according to the following formula: RPN = SE * PO * PD. The final RPN value for each event was between 1 and 1000. We have decided to set a limit for acceptance of a procedure if the sum of RPNs for all elements of the process was below 300. This limit was based on the data from application of FMEA methodology in industries other than healthcare. An industrial process, which reaches more than 300 RPN is considered unsafe, has to be stopped and should be restructurised or eliminated. Since the application of FMEA method in surgery is a relatively new concept, we could not find evidence from non-industrial setting for establishing a different RPN limit value.

After preparing the matrix for the process, we have designed a laparoscopy training in swine model programme based on FMEA methodology.

### Laparoscopic sigmoidectomy in a pig model

Four ports are placed for the laparoscopic sigmoidectomy in a swine model: (1) umbilical for the optics, (2) and (3) in the right flank for the surgeon’s hands and (4) in the left flank for the assistant. Surgical steps were as follows: opening of a mesenteric window and dissection of caudal mesenteric artery; identification of the left ureter; clipping of caudal mesenteric artery; mesorectal dissection and stapling of distal colon; cranial isolation of proximal segment of the colon; extracorporeal cutting of the specimen and placement of the anvil of a circular stapler; restoration of the pneumoperitoneum; end to end anastomosis; and checking for possible leakage.

### The laparoscopy training course

The training course was designed to last for three days. All participants were divided into teams of two surgeons. For each day of the course, every team disposed of one experimental animal. During the first meeting before entering the OR, the surgical anatomy of a pig and its differences from humans were exposed. Afterwards, the FMEA methodology was explained. Subsequently, the teams were invited to OR and were asked to perform laparoscopic sigmoidectomy maintaining oncology principles. During this first session, the students were not tutorized, i.e., they performed the sigmoidectomy according to their previous experience and technical skills. The tutors were present in the OR, but their role was limited only to record all failures and any technical help or instructions were prohibited. Once the first OR session was terminated, all failures committed by all teams were discussed and the calculation of RPN for each failure of each team was explained. If a calculated RPN for a team was higher than 300 points, it was interpreted that the procedure could not be performed in a human patient (a process with a RPN of more than 300 points is considered too dangerous to be preformed and should be eliminated). This phase of the course was dedicated to evidence evitable failures and create a Hawthorne effect (i.e., improvement in response to the fact of being studied) [9].

During the second session in the OR, the students received help from the tutors to eliminate the evitable failures as discussed during the meeting after the first OR session. After finishing the second OR session, the evitable failures of all teams were discussed in a similar manner.

During the third and last sessions in the OR, the students received help from the tutors as during the second session. After the third session, the RPN results of each team during the whole course were discussed.

### Follow-up

After completion of each course, a telephone follow-up was carried out for each participant. During the telephone interview performed 3 months after completion of the course, we have asked about the degree of implementation of the FMEA methodology and laparoscopic sigmoidectomy in their respective hospitals. We have specifically asked whether FMEA and laparoscopic approach have been implemented on regular basis or only in an anecdotal manner.

## Results

The three-day laparoscopic sigmoidectomy training course has been organized three times between 2010 and 2012 with 48 surgeons from Spain, Portugal and Poland participating. We have analysed the evolution of the RPN result of each team and each consecutive experimental animal (in three OR sessions, there were three experimental animals operated on by each team). The objective of the course was to teach each participant the ability to perform a laparoscopic sigmoidectomy in a non-supervised environment with a RPN result of less than 300 points.

To ensure the uniformity of the attendants at the course during application stage, previous level in laparoscopic colorectal surgery and experience in open colorectal surgery have been taken into account. Also, the future trainees were questioned about the possibility of implementing the laparoscopic technique in their respective hospitals. As a result, all attendees had between 6 and 10 years of experience in colon surgery, with 3–5 cases of open colorectal surgery per month or 1–2 cases of laparoscopic colorectal surgery per month. Further stratification of the participants based on their skills was not possible because of the limited group size.

**Fig. 1:**
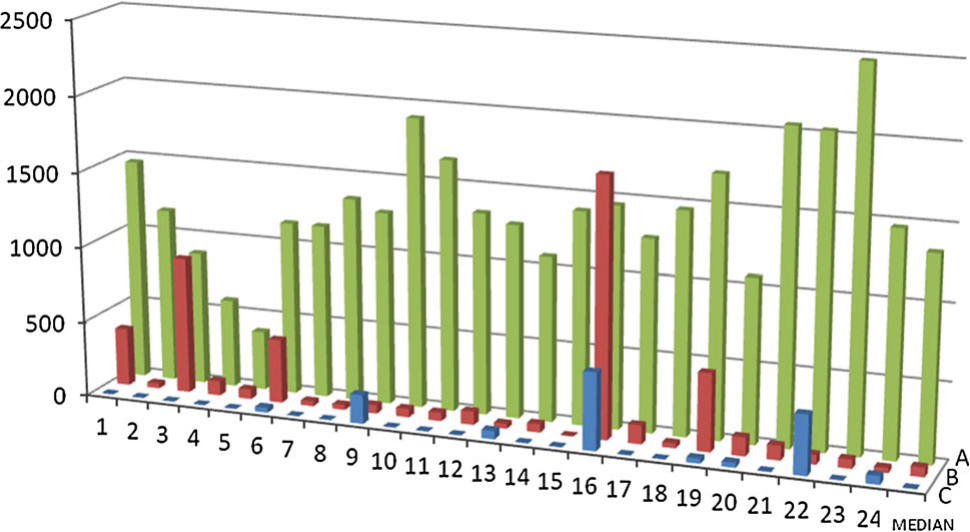
RPN results for each group for three consecutive operations for each OR session: *a* OR session 1, *b* OR session 2, *c* OR session 3. RPN—risk priority number

As we can see in Fig. 1, only three surgical teams out of 24 were able to obtain a RPN of less than 1000 points during the first session in the OR. However, the RPN result of each team had a tendency to decrease during the training course from a median RPN of 1339 ± 457 (first experimental animal) through 62 ± 381 (second experimental animal) to finally reach a median of 0 ± 130 (third experimental animal). Already during second OR session, only 5 teams received a RPN of more than 300 points. Interestingly, between the first and the second OR sessions, the students did not receive any technical training. As stated in the Methods section, during the discussion after the first OR session, we have only pointed out at which phases of the process the teams committed mistakes and how much those mistakes costed them in terms of RPN score. Also, the meaning of the final RPN result was clearly defined as acceptable or non-acceptable level of the risk for the patient. So, the decline in the RPN result during the second session was as the matter of fact only a result of the discussion about the failure mode and its implications for the patient. This effect was even stronger during the third OR session when only two teams were not able to receive a RPN score of less than 300 points.

**Fig. 2:**
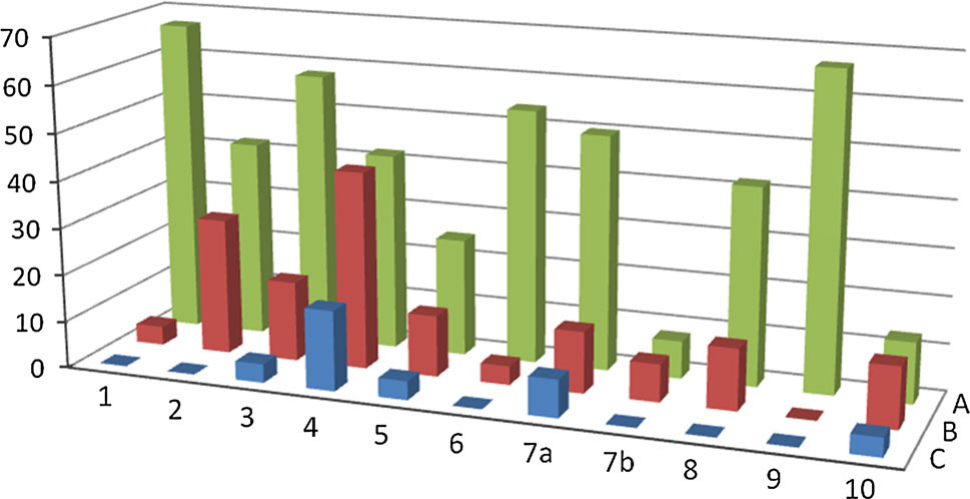
Percentage of surgeons committing failures during each phase for each OR session: *a* OR session 1, *b* OR session 2, *c* OR session 3. Surgical phase: *1* Fixation of patient to the operation table, *2* Trocar placement, *3* Obtaining surgical field, *4* Splenic flexure mobilisation, *5* Arterial pedicle dissection, *6* Ureter localization, *7a* Proximal specimen division, *7b* Distal specimen division, *8* Division of mesocolon, *9* Abdominal wall incision for specimen extraction, *10* Stapled anastomosis

In Fig. 2, we can observe the details of all types of failures committed by the trainees during all three OR sessions. We can observe a shift from the failures of proceeding type to the errors of technical type, the latter being the result of each participant laparoscopy skills prior to the course.

When analysing the type of most common errors committed by the teams during the first OR session, we have found that there were five phases of the process in which more than 50 % of the students committed mistakes. Those phases were as follows:

- positioning of the patient and fixation to the operating table (error: wrong position and bad fixation).
- positioning of the intestinal loops in order to gain operative field (error: not suitable surgical field).
- localisation of the ureter (error: lack of undoubtful localisation of the ureter).
- transection of the intestine (error: bad trocar positioning that leads to the use of too many stapler loads which favours anastomotic leaks).
- abdominal wall section for specimen extraction (error: laparotomy without previous exsufflation of the pneumoperitoneum which can result in “cancer spray” and cancer implants within the laparotomy scar).

All those errors fall into the class of proceeding-type errors. These type of errors typically do not depend on the technical skills of the person performing a task. The root of the proceeding-type errors is a protocol of action according to which a person is performing a task. Therefore, once those errors are identified they are relatively easy to eliminate; a new protocol should be implemented, and the team should know how important it is to stick to it.

During the second OR session, the errors were of technical type:

- bad trocar positioning.
- technical errors during splenic flexure mobilisation.

The last session in the OR was almost error-free. Only the mobilisation of the splenic flexure remained a difficult task that lead 17 % of surgeons to perform failures at this stage of the operation. As this part of laparoscopic sigmoidectomy is probably the most technically demanding, we have observed a relatively small decline in the percentage of surgeons failing to complete this step from 42 % during the first OR session. In contrast, all previously mentioned phases (proceeding-type errors) that posed important problems to more than half of the participants at the beginning of the course were completed in a correct manner by almost all surgeons at the end of the course.

During telephone follow-up performed 3 months after the completion of the course, all participants confirmed implementation of the FMEA methodology to laparoscopic sigmoidectomy in their respective hospitals. Laparoscopy has been used as a standard approach to all elective sigmoid colon resections except for patients with contraindication for the technique (as stated in the telephone interview).

## Discussion

The FMEA model has been tested in various clinical situations in healthcare. It was successfully applied to the management of blood transfusion risk [10], preventing errors in the radiology department [11] and management of drug prescription in the paediatric ward [12]. On the other hand, the efficacy of FMEA has been criticised in the literature for low accuracy [13]. Although its use is recommended by the United States Joint Commission as one of the proactive risk assessment procedures to be used in health care organizations [7], there exist only scarce reports on FMEA application in surgery [14]. We could not find in the literature any publication about the implementation of the FMEA principle to a laparoscopy surgery training.

The wide acceptance of laparoscopy techniques in surgery leads to the need of a training programme that would prepare surgical trainees in a best possible way to perform laparoscopy procedures. The results of several type of training programmes that use simulators, experimental animals or human cadaveric models are encouraging [3]. On the other hand, the technical skills learned during those programmes are relatively quickly lost by participants if they do not continue to use the newly learned skills after the course [2]. Also, we still do not know what kind of learning environment is ideal for the best absorbent of the laparoscopy skills [2–4]. It is also not clear whether laparoscopy training should be done individually or with a tutor [15].

In our study, the failure modes during laparoscopic sigmoidectomy with highest RPNs (i.e., with the most serious danger for a patient) during the first evaluation came from five mistakes relatively easy to avoid: bad positioning with bad fixation of the patient, bad surgical field, no ureter localisation, too many stapler loads used for intestinal transection and incision for specimen extraction without previous exsufflation. As observed in our study, a simple analysis of these failure modes resulted in an important decrease in the RPN results. The difference observed between the first and the second OR sessions in our study can be therefore attributed almost entirely to the Hawthorne effect [9]. Contrary to FMEA methodology, in case of quantitative and qualitative assessment tools (e.g. OSATS) which are more frequently used to evaluate progress in surgical laparoscopy training, the focus is set almost entirely on correcting technical errors of the trainees. Therefore, since our methodology was not concentrating on technical aspects of the process (laparoscopic sigmoidectomy), the technical errors remained present throughout the course.

Colon laparoscopy requires important changes in patient position during surgery to obtain good exposure. Bad fixation of the patient to the operating table can result in falling of the anaesthetised patient from the table which is an extremely serious event. It turned out that it is sufficient to stress how disastrous consequences can have bad patient fixation to practically eliminate this failure mode from the subsequent OR sessions. The same was true for other protocol-related failure modes like obtaining surgical field, localization of the ureter and abdominal wall incision for specimen extraction.

Technical skills-related failure modes are more time consuming to eradicate, as they require a lot of training for the students to master and maintain these skills. However, as we have shown in our study, a simple discussion about the failure modes can result in a sharp decrease in proceeding-type errors and lead to an acceptable RPN result. This in turn allows the trainees to perform the trained operation relatively safe on a human patient even if their technical skills are not perfect. This finding is quite important since the learning curve in colon laparoscopy can take as long as 87–152 operations [16].

Our findings also underline the need for regular team meetings before surgery during which the failure modes of each surgery should be explained to all team members [17]. It is quite clear that the successful implementation of the FMEA is dependant on the aptitude of the team members to hold regular meetings [10].

Currently, there is no prospective risk assessment method that provides an absolute safety to high-risk health care procedures. The systematic application of the FMEA in non-surgical settings can give positive results already one year after its implementation [18]. It is our opinion that the implementation of this system in a systematic manner to the laparoscopic surgery training can contribute to reduce the risk of human error and thus improve patient safety.

Proactive risk assessment methods can be successfully used to address safety in surgical ward by considering risk associated with all activities of a surgical ward [19]. As we have shown in this paper, the same proactive approach can be introduced to surgical laparoscopy course.

## Conclusion

The FMEA can be successfully implemented to laparoscopy training and can help eradicate non-technical surgical errors.
